# Molecular Insights into Determinants of Translational Readthrough and Implications for Nonsense Suppression Approaches

**DOI:** 10.3390/ijms21249449

**Published:** 2020-12-11

**Authors:** Silvia Lombardi, Maria Francesca Testa, Mirko Pinotti, Alessio Branchini

**Affiliations:** Department of Life Sciences and Biotechnology, University of Ferrara, 44121 Ferrara, Italy; lmbslv@unife.it (S.L.); mariafrancesca.testa@unife.it (M.F.T.)

**Keywords:** premature termination codons, nonsense mutations, ribosome readthrough

## Abstract

The fidelity of protein synthesis, a process shaped by several mechanisms involving specialized ribosome regions and external factors, ensures the precise reading of sense and stop codons. However, premature termination codons (PTCs) arising from mutations may, at low frequency, be misrecognized and result in PTC suppression, named ribosome readthrough, with production of full-length proteins through the insertion of a subset of amino acids. Since some drugs have been identified as readthrough inducers, this fidelity drawback has been explored as a therapeutic approach in several models of human diseases caused by nonsense mutations. Here, we focus on the mechanisms driving translation in normal and aberrant conditions, the potential fates of mRNA in the presence of a PTC, as well as on the results obtained in the research of efficient readthrough-inducing compounds. In particular, we describe the molecular determinants shaping the outcome of readthrough, namely the nucleotide and protein context, with the latter being pivotal to produce functional full-length proteins. Through the interpretation of experimental and mechanistic findings, mainly obtained in lysosomal and coagulation disorders, we also propose a scenario of potential readthrough-favorable features to achieve relevant rescue profiles, representing the main issue for the potential translatability of readthrough as a therapeutic strategy.

## 1. Ribosome Fidelity and Translation Termination

The translation of mRNA is a ribosome-catalyzed process leading to the synthesis of a polypeptide chain driven by the correct base pairing between mRNA codons and aminoacyl-tRNAs (aa-tRNAs) anticodons [1,2]. This biological event consists of three main phases, namely initiation, elongation, and termination.

**Figure 1:**
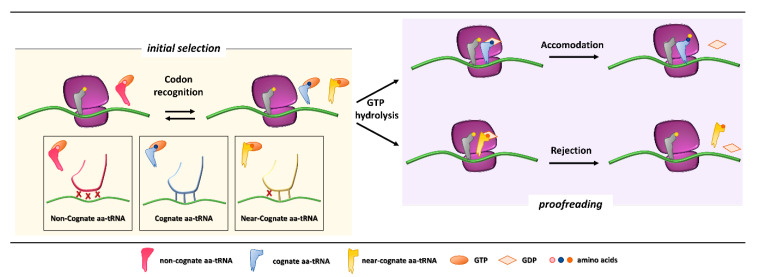
Initial selection and proofreading activity of the translating ribosome. The insertion of cognate or near-cognate, as well as the rejection of non-cognate, aa-tRNAs is exerted by the ribosome during the initial selection step (**left panel**, with codon-anticodon interactions depicted in the boxes below). Upon GTP hydrolysis, the cognate aa-tRNA is efficiently accommodated, with formation of peptide bond and progression of protein synthesis with a new aa-tRNA selection step (**right panel**, **upper part**). The presence of a near-cognate aa-tRNA, which fails to be accommodated, results in rejection of the aa-tRNA through a proofreading step (**right panel**, **lower part**).

In the first phases of the translation process, interaction among mRNA, the initiator tRNA (placed in the ribosome P site), translation initiation factors, and the small ribosome subunit leads to the recruitment of the large subunit and formation of the initiation complex. Then, a process of aa-tRNA sampling occurs at each mRNA codon during the elongation phase. Each aa-tRNA enters the ribosomal A site as a ternary complex with a GTPase elongation factor (EF-Tu in prokaryotes and eEF1A in eukaryotes) and a GTP molecule. During this phase, the ribosome discriminates with high fidelity the cognate from non-cognate or near-cognate ternary complexes through two strategies, namely initial selection and proofreading. Both steps, separated by the irreversible hydrolysis of GTP, rely on the different stability of codon-anticodon matches [3,4] (Figure 1).

Initial selection allows for an efficient rejection of non-cognate aa-tRNAs on the basis of two out of three mismatches in the codon-anticodon duplexes, which leads to the dissociation of the incorrect aa-tRNA ternary complex with no costs in terms of GTP [5]. This kinetic mechanism alone is not enough to distinguish between cognate and near-cognate aa-tRNA complexes. Indeed, interactions of decoding center elements, located within the prokaryotic (16S) or eukaryotic (18S) rRNA, with both mRNA codon and tRNA anticodon are necessary to increase ribosome accuracy [6,7]. The ribosome fidelity during translation has been described in prokaryotes, but it is similar in eukaryotes. In particular, when a ternary complex enters the ribosomal A site, the binding of a cognate aa-tRNA anticodon to a mRNA codon induces two conserved adenines, A1492 and A1493 (prokaryote numbering), to flip out of an internal loop of helix 44 in 16S rRNA. Moreover, also the universally conserved G530 switches from the *syn* to the *anti*-conformation. This new arrangement enables the two key adenines to interact through hydrogen bonds with the first and second base pairs of the codon-anticodon helix, while the G530 interacts with the second anticodon position and the third codon position. As a result, the induced changes lead to the discrimination between correct Watson–Crick geometry, due to standard base pairing, at the first and second codon positions, whereas the third “wobble” base pair may accommodate other non-standard geometries [8,9]. Therefore, the recognition of the cognate aa-tRNA causes a local conformational change in the decoding site eliciting a transition from an open to a closed form of the small ribosomal subunit ensuring the subsequent hydrolysis of GTP [9]. Although it has been defined how recognition of a cognate aa-tRNA occurs, it is still unclear what the entry of near cognate aa-tRNA in ribosomal A site entails. One hypothesis is that the decoding site does not sense a correct Watson–Crick codon-anticodon base pairing, with the lack of stabilization for the near-cognate complex and its preferential rejection. Furthermore, structural studies reveal that binding of a near-cognate aa-tRNA does not induce the closed conformation of the small subunit, which rather remains in an open or partially-closed conformation [10]. However, it seems quite clear that a near-cognate aa-tRNA escapes from the initial selection step when a mismatch in the codon-anticodon helix mimics a Watson–Crick geometry [11,12,13].

After codon recognition, which leads to the closed conformation of the small ribosomal subunit, a series of rearrangements occur in the aa-tRNA, leading to activation of the elongation factor GTPase. The hydrolysis of GTP consists of the docking of the elongation factor GTPase into the sarcin-ricin loop (SRL) within the large ribosomal subunit [8]. It has been demonstrated that, through local conformational changes in the small ribosomal subunit, the binding of a cognate aa-tRNA accelerates GTP hydrolysis in comparison with a near-cognate aa-tRNA [14,15]. Following the detachment of the GDP-bound elongation factor, an accommodation step of the aa-tRNA takes place, the mechanism of which can explain how the aa-tRNA proofreading occurs. The aa-tRNA moves into the peptidyl-transferase center, located within the large ribosomal subunit, through reversible fluctuations of its elbow region, acceptor arm and 3′CCA end from A to P site [15,16]. It seems that a cognate aa-tRNA accommodates more rapidly than a near-cognate aa-tRNA due to a more stable codon-anticodon base pairing during the proofreading step. On the other hand, unfavorable Watson–Crick geometry and weak codon-anticodon interactions probably facilitate rejection of a near-cognate aa-tRNA, which circumvented the initial selection step [12,17]. Overall, ribosome fidelity strategies strictly monitor the aa-tRNA selection during the translation process.

The final phase, translation termination, is triggered when a stop codon (UAA, UAG, UGA) enters the ribosomal A site [18,19]. In general, extra-ribosomal proteins, named release factors (RFs), are recruited to promote the release of the nascent polypeptide [20]. In eukaryotes, an essential role is played by the two main effectors eRF_1_ and eRF_3_. The eRF_1_ protein consists of three domains, namely an N-terminal domain directly recognizing all stop codons, a middle domain containing a conserved GGQ (Gly-Gly-Gln) motif involved into stimulation of polypeptide chain hydrolysis, and a C-terminal domain that binds eRF_3_ [21]. The second release factor, eRF_3_, consists of different domains, among which the most relevant is the G domain, which binds GTP and assists the termination process through GTP hydrolysis [22]. Similar to the elongation phase, when a stop codon enters the ribosomal A site, a process of aa-tRNA ternary complex sampling occurs. At the same time another ternary complex, composed of eRF_1_, eRF_3,_ and GTP, competes for stop codons recognition [23], driven by the N-terminal domain of eRF_1_ that establishes multiple contacts with the 40S subunit. The key step of GTP hydrolysis, prevented by the middle domain of eRF_1_ during formation of the pre-termination complex, is triggered by interactions involving eRF_3_ and the SRL [24]. Upon GTP hydrolysis, the positioning of eRF_1_ GGQ motif into the peptidyl transferase center stimulates the hydrolysis of the ester bond between tRNA and the polypeptide chain [25].

## 2. Nonsense Mutations and mRNA Quality Control

A relevant detrimental effect on protein synthesis may be exerted by nonsense mutations, which account for 11% of all gene alterations responsible for inherited human genetic diseases [26]. Nonsense mutations are defined as single-base pair substitutions affecting gene coding regions, which convert an mRNA sense codon into an in-frame premature termination codon (PTC). The impairment of gene expression due to PTC-containing transcripts is related to (i) the degradation of truncated proteins, with decreased stability or loss-of-function features, resulting from premature termination of translation, or (ii) the reduction in the steady-state level of cytoplasmic mRNA by the nonsense-mediated mRNA decay (NMD) quality control system [27,28,29]. However, it has been demonstrated that 5–25% of aberrant mRNAs might escape NMD, thus producing a truncated protein that, in autosomal disorders, might interfere with the wild-type protein function and give rise to dominant-negative effects [30].

**Figure 2:**
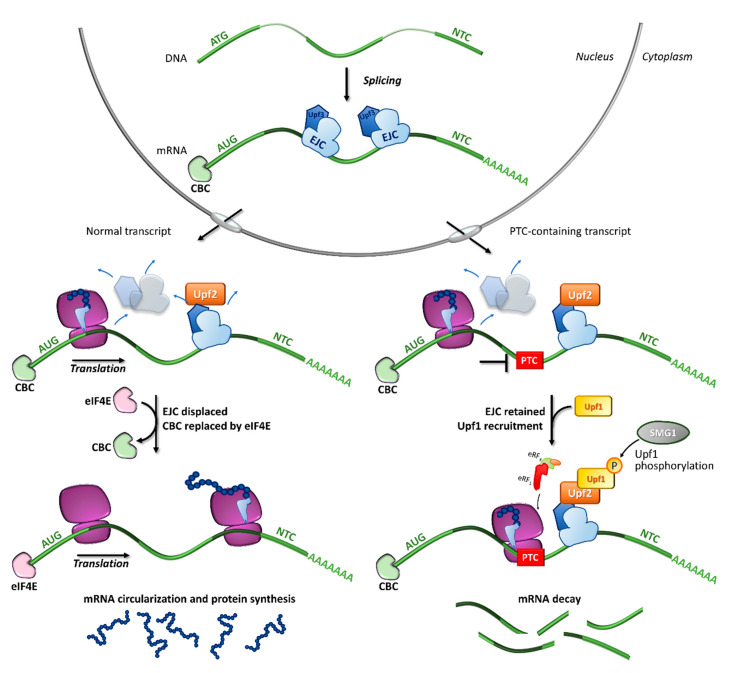
Fates of normal or PTC-bearing mRNA transcripts during the first step of translation. The splicing of pre-mRNA into the nucleus results in a mature mRNA transcript bound to exon-junction complexes (EJCs) as well as other key components such as the Upf3 (in the nucleus) and Upf2 (in the cytoplasm) proteins. Once transported into the cytoplasm, the mRNA undergoes a first (pioneer) round of translation. In normal conditions, the ribosome displaces all EJCs, resulting in the replacement of CBC with eIF4E, mRNA circularization and protein synthesis, which proceeds until the natural termination codon (NTC) is reached. If a premature termination codon (PTC) is present, EJCs are not efficiently removed by the ribosome, resulting in recruitment of eRF_1_, eRF_3_, Upf1 and the SMG1 kinase, which leads to Upf1 phosphorylation and degradation of the UPF1-tagged mRNA.

In particular, the NMD mechanism degrades PTC-bearing transcripts through the interplay of *cis* and *trans* factors. The first signal is provided by the exon junction complexes (EJCs), a multiprotein set deposited ∼20–24 nucleotides upstream of each exon-exon junction as a marker of the occurrence of splicing events [31,32,33]. It has been shown that PTC-bearing mRNAs bound to the cap-binding complex (CBC, consisting of CBP80-CBP20) can be recognized as NMD substrates during the so-called pioneer round of translation, namely a first translational cycle in which the ribosome performs an mRNA scan. During the pioneer round of translation, if the stop codon is located at the 3′ of the coding region, typical of natural stop signals, the ribosome displaces all EJCs, which allows the subsequent replacement of CBC with eIF4E and makes the mRNA immune to NMD. Conversely, if the transcript harbors a PTC, causing ribosome stalling, the NMD pathway is engaged due to the presence of downstream EJCs that cannot be removed [34]. Accordingly, immuno-purification studies showed that eIF4E-bound mRNAs were not associated with factors required for NMD, thus supporting that NMD takes place during the pioneer round of translation [35] (Figure 2).

Among the proteins involved in mRNA decay, the major orchestrator is Upf1, an ATP-dependent RNA helicase that is recruited on the PTC together with eRF_1_, eRF_3_, and SMG1 (phosphatidylinositol 3-kinase-related protein kinase) in the so-called SURF (SMG1-Upf1-eRF_1_-eRF_3_) complex [36]. Other components are required for the NMD activation, such as Upf3 or 3X, which are generally associated with EJCs in the nucleus, and Upf2, which is assembled to EJCs after mRNA export to the cytoplasm [35,37,38]. Once the PTC-bearing transcript is identified by the NMD machinery, several rearrangements and conformational changes occur, including the Upf1-mediated formation of the decay-inducing complex formation (DECID) [39,40], which leads to degradation of the tagged mRNA in a process driven by phosphorylated Upf1 [41,42]. Finally, the NMD factors are disassembled by Upf1, which in turn is converted to its unphosphorylated form [43].

## 3. Ribosome Readthrough

### 3.1. Mechanism, Programmed Readthrough and Natural vs. Premature Stop Codons

**Figure 3:**
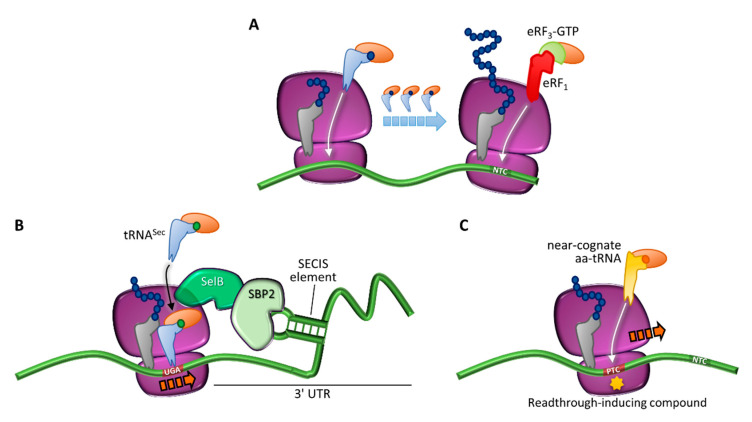
Translation termination and programmed or drug-induced PTC readthrough. (**A**) Translation in normal conditions, driven by incorporation of cognate aa-tRNAs, and termination elicited by eRF_1_ and eRF_3_ at the natural termination codon (NTC). (**B**) Programmed readthrough, as a result of terminal UGA recoding through incorporation of a selenocysteine-carrying aa-tRNA (tRNA^Sec^) mediated by a specialized elongation factor (SelB) and a downstream hairpin structure (selenocysteine inserting sequence, SECIS) recognized by the SECIS-binding protein 2 (SBP2). (**C**) Readthrough-mediated suppression of a PTC through incorporation of a near-cognate aa-tRNA, which can occur spontaneously or be induced by compounds.

The central role of protein synthesis has forced cells to develop accurate mechanisms to avoid translational errors, but termination, although effective, is not 100% efficient. As described above, during translation termination a competition for stop codon recognition occurs (Figure 3A). Normally, release factors highly outcompete aa-tRNAs, thus driving polypeptide chain release. However, in rare cases, a stop codon can be decoded by an aa-tRNA so that an amino acid is added to the polypeptide chain and elongation proceeds until the next in-frame stop codon is reached [44]. This mechanism is called ribosome readthrough and can be mediated by a near-cognate aa-tRNA (i.e., a tRNA whose anticodon is complementary to two out of the three positions of a stop codon), or by specialized tRNAs carrying the 21st and 22nd amino acids pyrrolysine and selenocysteine [45,46] (Figure 3B). In particular, pyrrolysine is encoded by the UAG stop codon and is restricted to several microbes, whereas selenocysteine is encoded by the UGA codon and can be found in bacteria, archaea, and eukaryotes. Notably, the incorporation of selenocysteine requires a specific insertion sequence (selenocysteine inserting sequence, SECIS) and is necessary for the synthesis of selenoproteins [45,46].

Ribosome readthrough was first identified in viruses as a way to expand their genetic information. It was observed that in *Escherichia coli* infected by RNA phage Qβ, the tRNA^Trp^ stimulated readthrough over the UGA stop codon at the end of the coat protein cistron, resulting in a longer coat protein essential for the production of infective Qβ particles [47]. Since then, stop codon readthrough has been found to play important biological roles in several organisms, providing a mechanism to regulate gene expression through the production of different proteins from the same gene [48,49]. This phenomenon has been called programmed readthrough and its prevalence varies among organisms, with a few examples found also in humans [44,50,51].

Nevertheless, the readthrough of natural stop codons is generally a rare event and the termination signals at the end of an open reading frame evolved several ways to promote efficient termination, especially among highly expressed transcripts. A paradigmatic example is represented by tandem stop codons, which act as “back up” termination signals in case of readthrough [52,53]. Moreover, the nonstop decay surveillance mechanism recognizes and degrades those transcripts with stalling ribosomes bound to the poly(A) tail, thus preventing the generation and accumulation of unwanted C-terminally extended proteins [54]. In addition, recent studies suggested that translation into 3′ UTRs might reduce C-terminally extended protein levels through 3′ UTR-encoded peptides that destabilize the attached protein, either co- or post-translationally [55].

This scenario changes in the presence of a PTC, inserted for instance by a nonsense mutation. In this case, the frequency of readthrough is about 10-fold higher (0.01–1%) than at natural stop codons (0.001–0.1%) [56,57,58,59], likely due to the interaction between the termination complex and factors bound to the 3′ untranslated region (UTR) of the mRNA. Indeed, during translation, the mRNA is maintained in a closed-loop conformation whose function is both to protect the ends of the transcript from exonucleolytic degradation [60], and to enhance recycling of translational components, thus increasing the frequency of translation initiation. This closed conformation is maintained by the association of three actors, namely the cap-binding protein eIF4E, which is bound to the 5′-cap structure of the mRNA, the poly(A)-binding protein (PABP), attached to the poly(A) tail, and eIF4G, which binds both eIF4E and PABP resulting in mRNA circularization. Along with its role in the formation of the closed-loop complex, PABP also promotes translation termination by interacting with eRF_3_ and stimulating polypeptide chain release [61]. In the presence of a PTC, which is usually distant from the poly(A) tail, the interaction between PABP and eRF_3_ will likely be less efficient, leading to prolonged ribosomal pausing and increased aa-tRNA sampling, which in turn makes the PTC more susceptible to readthrough [62].

### 3.2. Readthrough-Inducing Compounds

It has been widely demonstrated that the readthrough-mediated suppression of PTCs is promoted by the presence of certain small molecular weight compounds [63], thus highlighting their potential application to treat diseases caused by nonsense mutations (Figure 3C).

The first attempt to use readthrough inducing molecules for therapeutic purposes dates back to 1996, when Howard and colleagues showed that nonsense mutations in the *CFTR* gene could be suppressed by the aminoglycoside G418 (geneticin), as demonstrated by the appearance of full-length functional proteins in a cystic fibrosis model system [64]. Since then, therapeutic applications of readthrough-inducing compounds have been widely explored in several disease models, from in vitro reporter systems [57,65], to patient-derived cells [66,67], animal models [68,69], and clinical trials [70,71].

**Figure 4:**
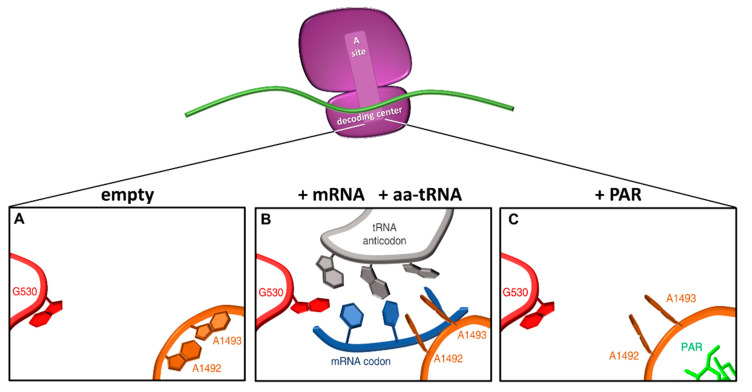
Conformational changes in the decoding center upon tRNA or aminoglycoside binding. Schematic representation of the ribosomal decoding center as derived from PDB 1IBM (**A**), 1IBL (**B**), and 1IBK (**C**) (adapted from [1]). In the native 30S ribosomal subunit, adenines 1492 and 1493 are stacked inside helix 44, and G530 in the *syn* conformation (**A**). When the correct codon-anticodon loop, formed upon entering of a cognate aa-tRNA, enters the A site, the A1492 and A1493 residues flip out and G530 switches to the *anti*-conformation (**B**). In the presence of paromomycin (PAR), the A1492 and A1493 residues are similarly flipped out into the A site mimicking the presence of a cognate aa-tRNA. Red, G530 loop; orange, helix 44; grey, tRNA anticodon; blue, mRNA codon; green, paromomycin.

The first and most studied readthrough-inducing molecules are aminoglycosides, a class of structurally related antibiotics characterized by a common 2-deoxystreptamine core (ring II) linked to a glucopyranosyl (ring I) at position 4. Aminoglycosides exert their antibiotic activity by binding to the bacterial ribosome and inhibiting protein synthesis [72]. In particular, they bind to specific nucleotides in the decoding center inducing A1492 and A1493 to flip out from the internal loop of helix 44, a conformational change that partially resembles that caused by cognate aa-tRNA binding (Figure 4), thus resulting in extensive amino acid misincorporation and translation inhibition [1]. In humans, aminoglycosides can be safely used as antibiotics because their binding to the eukaryotic ribosome is much less efficient. Extensive mutational analyses revealed that the major determinants of the different aminoglycoside sensitivity between prokaryotes and eukaryotes are the yeast G1645 and A1754 residues, corresponding to *Escherichia coli* A1408 and G1491 residues. These divergent nucleotides cause the aminoglycoside binding pocket within helix 44 to be shallower in eukaryotes, thus preventing stable insertion and binding of the drug [72]. Nevertheless, in the presence of a PTC, the impact of aminoglycosides on the eukaryotic ribosome is sufficient to reduce discrimination between near-cognate aa-tRNAs and release factors, thus enhancing readthrough [57,73].

The molecular basis of aminoglycosides effects is still unclear, but recent structural analysis of yeast ribosomes in a complex with different aminoglycosides revealed that these compounds interact with the eukaryotic ribosome at multiple sites and that the mechanism by which each aminoglycoside induces PTC readthrough is specific [74]. In particular, aminoglycosides containing 6′-OH substituent in ring I (e.g., G418) bind to the pocket within helix 44, thus facilitating near-cognate aa-tRNA accommodation and competition with the release factor. On the other hand, aminoglycosides containing a 6′-NH_2_ substituent (e.g., gentamicin) employ an alternative mechanism based on inter-subunit rotation effects that are likely to hamper the interaction between release factors and the ribosome [74]. In the context of a nonsense suppression therapeutic approach, however, the lifelong administration of aminoglycosides is hardly feasible because their use is associated with persistent and serious ototoxicity and nephrotoxicity [75,76]. The mechanism underlying this toxicity is currently unclear, but generation of reactive oxygen species is thought to be the principal cause of cell death [77].

Several approaches have been investigated to overcome aminoglycosides toxic effects, such as liposome encapsulation [78] and co-administration with antioxidants [79] and other compounds able to reduce the interaction with cellular components [80] or to increase the readthrough-inducing activity of aminoglycosides [81]. In addition, a different approach stemmed from the idea that distinct structures of aminoglycosides are responsible for nonsense suppression properties and toxicity. Therefore, aminoglycosides have been chemically modified to reduce toxicity and increase the therapeutic potential. In particular, the paromomycin derivatives NB30 and NB54 showed up to 15-fold reduced toxicity [82], whereas the G418 derivatives NB74 and NB84 showed reduced toxicity, increased activity and the ability to cross the blood-brain barrier in mice [69]. Moreover, NB124 was shown to reduce NMD-mediated degradation of the target transcript [83], and the neomycin derivative pyranmycin (TC007) was able to increase the lifespan of spinal muscular atrophy mice [84].

A different approach based on high-throughput screening of small molecular weight compounds libraries led to the identification of non-aminoglycoside molecules with readthrough-induction activity [85,86,87]. One paradigmatic example is PTC124 (also known as ataluren or Translarna^®^), an orally bioavailable oxadiazole compound with PTC suppression activity that has been shown to be safe, with minimal off-target side-effects and no antibacterial activity [85]. Although the mechanism of action of PTC124 has yet to be clarified, encouraging results have been obtained. Different diseases are currently being evaluated in clinical trials, but results are conflicting. Indeed, ataluren was recently approved by the European Medicines Agency (EMA) for Duchenne muscular dystrophy patients with nonsense mutations [71,88], whereas the lack of conclusive results obtained in pre-clinical and clinical trials led to discontinuation of the development of ataluren for cystic fibrosis [70]. Other promising readthrough inducing compounds recently identified include RTC13 and RTC14 [86], amlexanox [89], PTC414 [90], 2,6-diaminopurine [91], and ELX-02 [92,93], with the latter currently under clinical investigation [94].

### 3.3. The Determinants of Readthrough

The establishment of therapeutic approaches based on nonsense suppression has been hampered to date by their extremely variable outcome. Indeed, the efficacy of readthrough induction depends on several factors, including the amount of target transcript, the efficiency of PTC suppression, and the features of the full-length proteins arising from a readthrough event.

#### 3.3.1. The Amount of Target Transcript

The amount of target transcript is related to the extent of NMD and to the occurrence of aberrant splicing. As mentioned above, the NMD surveillance mechanism downregulates PTC-bearing transcripts, thus preventing the synthesis of truncated and potentially damaging proteins [41,95]. However, in the context of a PTC suppression approach, NMD occurrence can affect the efficacy of readthrough by reducing the availability of its substrate. For this reason, combined approaches that promote readthrough and inhibit NMD have been tested, resulting in increased PTC suppression [96]. Moreover, transcripts with a nonsense mutation causing aberrant splicing that results in the removal of the coding region harboring the PTC from the mature mRNA (i.e., exon skipping) are expected to be less responsive to readthrough due to reduced levels of the correctable target [97]. This possibility should be also considered when predicting or interpreting the readthrough induction outcomes.

#### 3.3.2. The Sequence Context

The PTC sequence context, namely the stop codon present and the surrounding sequences, is a major determinant of readthrough efficiency [98]. It is widely established that the three stop codons are decoded with different accuracy by the ribosome, and are thus differently susceptible to readthrough with the rank order of UGA ≥ UAG > UAA [57,99,100]. However, early statistical studies of natural termination sequences had highlighted a bias in the nucleotide immediately following the stop codon (+4 position, with the first nucleotide of the termination codon marked as +1), suggesting that the actual termination signal is a tetranucleotide [101]. Indeed, later studies confirmed that the +4 position is strongly involved in termination fidelity and readthrough efficiency, with an up to 6-fold difference conferred solely by the 4th nucleotide [57,102]. Structural studies of the eukaryotic ribosomal complex containing eRF_1_ revealed that when a stop codon is placed in the ribosomal A site, the base at position +4 is pulled into the A site by mRNA compaction, where it is stabilized by stacking against G626 of 18S rRNA [101,103,104,105]. The stacking of G626 is less stable for pyrimidines, which help explaining the readthrough-favorable role of cytosine and uracil at the +4 position. Indeed, extensive studies in yeast showed that the UGAC and UAGU signals are the most efficient in mediating readthrough, followed by UGAN and UAGN, whereas the UAAN signals, and in particular the UAAA sequence, mediate the most accurate termination [57].

Besides the tetranucleotide termination signal, both 5′ and 3′ surrounding sequences (from −2 to +9 positions) showed a non-random distribution of nucleotides in natural contexts [56]. Concerning the 5′ sequence, the identity of the two nucleotides immediately upstream of the stop codon showed a significant effect on readthrough efficiency and, in particular, two adenines in positions −2 and −1 were associated to the highest levels of readthrough [106,107]. In addition, the 3′ sequence context modulates termination fidelity, with a significant but complex role of the +5, +6 and +8 nucleotides. Interestingly, this effect is more prominent in the presence of the leakiest stop codon (UGA), whereas UAG and UAA codons are less influenced by the downstream sequence [102]. The structural basis for these effects is unclear, although the P site codon (including positions −2 and −1) appears to directly contact the top of helix 44 of 18S rRNA [107], and the +4 and +5 nucleotides to stack with G626 and C1698 bases of 18S rRNA [104], respectively. This highlights the presence of a complex network of interactions between ribosome and the sequences surrounding the stop codon, although the effects of 5′ and 3′ sequences are subtle and subordinated to the stop codon present.

Overall, readthrough efficiency is hardly predictable based strictly on the sequence context but most authors agree on the high susceptibility of the UGA stop codon followed by UAG and UAA codons, the latter being the less responsive. Notably, the levels of readthrough induced by several aminoglycosides seem to be simply superimposed on the basal level of readthrough allowed by each sequence context [57], and this should be considered to identify more susceptible nonsense mutations in the context of a PTC suppression approach, although experimental evidence is always desirable.

#### 3.3.3. The Reinserted Amino Acid

Another critical but often underestimated factor affecting the readthrough outcome is the amino acid reinserted at the PTC position. As mentioned above, readthrough occurs when a stop codon is misrecognized by the ribosome, which incorporates an aa-tRNA instead of terminating translation. Extensive studies in yeast and mammalian expression systems have shown that just a defined subset of aa-tRNAs is actually used by the ribosome during readthrough, with UAA and UAG codons that can be suppressed by either glutamine, tyrosine or lysine, whereas UGA codons can be decoded by tryptophan, arginine, or cysteine [65,108]. Interestingly, the comparison of several studies showed that, even though the set of inserted amino acids is the same, different experimental systems and readthrough-inducing compounds can affect the insertion frequencies of the different amino acids at a specific PTC [65,108,109]. In particular, a comprehensive study performed in yeast by Roy and colleagues showed that different readthrough-inducing conditions such as loss of Upf factors, defective release factors, or the presence of aminoglycosides, were all able to change the proportion of amino acids reinserted during readthrough [65].

The structural basis for aa-tRNA selection has been partly unveiled. Recent studies showed that the ribosomal decoding center tolerates nonstandard Watson–Crick base pairs in the A site, with the shape of the base pair being crucial, rather than the number of hydrogen bonds formed [110]. In particular, the aa-tRNA insertion at a PTC occurs by mispairing at either position 1 or 3 of the stop codon. The UAG codon showed a preference for mismatches at position 1, whereas UGA favoured mispairings at position 3, and UAA showed equal distribution at both positions [65]. Moreover, the identification of the amino acids reinserted during readthrough suggested that, at the third codon position, the A-C mispairing is favored over A-G and G-G, whereas in the first position the U-G mispairing is predominant, likely due to its geometrical mimicry of a standard base pairing [65,110,111].

Importantly, the amino acids reinserted during PTC readthrough dictate the features of the resulting full-length proteins, and thus represent a crucial determinant of the outcome resulting from a nonsense suppression approach. Indeed, the result of readthrough is an assortment of full-length proteins with different missense changes at the site of the PTC, which can have major impact on protein stability and/or function. For instance, the reinsertion of the wild-type residue or a tolerant non-conserved position would favour the production of a functional protein. On the other hand, if the PTC affects an essential position (e.g., a catalytic residue), the protein arising from readthrough would be full-length but probably non-functional. Therefore, careful evaluation of each PTC should be performed to drive the selection of nonsense mutations amenable for suppression.

Overall, the specific features of each PTC (sequence context and role of the residue in the protein) should be carefully considered in order to help predicting and interpreting the results of a nonsense suppression approach.

## 4. Implications for a Nonsense Suppression Approach

The ability of some drugs to interact with the ribosomal decoding center and to alter translation fidelity has shed light on the therapeutic potential of the readthrough-inducing approach to suppress nonsense mutations and lead to the synthesis of full-length proteins [108,112,113].

Since the first attempt by Howard and colleagues in 1996 [64], several relevant diseases have been challenged with this correction strategy, including Duchenne/Becker dystrophy [68,114,115,116,117,118], cystic fibrosis [119,120,121,122,123,124], and spinal muscular atrophy [84,125,126]. Broad information on the efficacy of drug-induced readthrough for these disease models, as well as a detailed discussion on readthrough -inducing compounds, have been recently reviewed [87,127,128,129]. In addition, further details on approaches related to stop codon suppression or other strategies for the correction of molecular defects in genetic disorders have also been described [130,131,132].

**Figure 5:**
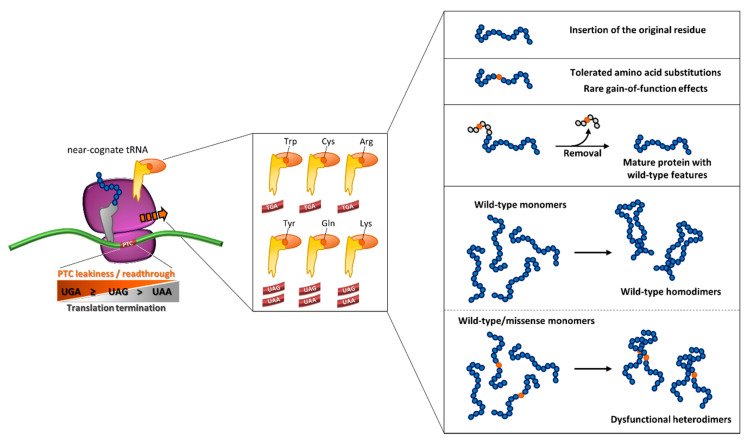
Outcome of readthrough as an integrated interplay of molecular determinants. The degree of readthrough of translating ribosome is driven by PTC type, which leads to the differential insertion of a subset of amino acids brought by near-cognate aa-tRNAs. A scenario of the resulting readthrough-mediated protein output is depicted in the right panels (see text for details).

Notably, a relevant body of work has focused on the qualitative or final output of readthrough (e.g., through reporter assays), mainly considering the nucleotide sequence context as the major driving force. However, the underlying complexity of this process would benefit from an integrated experimental approach on the molecular determinants dictating the resulting protein output. In this scenario, the prediction of readthrough-favorable features and models to characterize protein variants bearing predicted amino acid insertions, may shed light on those mutations being eligible for treatment with readthrough-promoting drugs (Figure 5).

These considerations, which are pivotal for potential therapeutic purposes, should also include two additional elements, namely (i) whether the readthrough approach is compatible with the estimated threshold needed to ameliorate the disease phenotype, and (ii) the possibility that, for proteins involved in the formation of oligomers, missense variants arising from readthrough could exert dominant-negative effects. These elements have been mainly detailed in metabolic and coagulation disorders as models.

### 4.1. Readthrough in Lysosomal Storage Disorders

Lysosomal storage disorders (LSDs) are a group of rare inherited metabolic diseases associated with deficiencies in key metabolic enzymes and characterized by the accumulation of undegraded substrates within lysosomes in multiple tissues and organs [133].

A correction approach based on drug-induced readthrough could be particularly relevant for LSDs patients, due to the relatively low threshold necessary to potentially alleviate the disease phenotype [134]. In addition, nonsense mutations are relatively frequent in LSDs, particularly in Mucopolysaccharidosis I-Hurler (MPS I-H), in which a high proportion of patients (50–70%) is estimated to be affected by a nonsense mutation [23].

#### 4.1.1. The Model of MPS I-H

MPS I-H (OMIM #607014) is the severe form of α-L-iduronidase (IDUA) deficiency and is associated with glycosaminoglycan (GAG) accumulation [135]. In this disease, the Q70X and W402X are the two most common IDUA nonsense mutations in Caucasians [136,137].

Constructs bearing either the W402X (UGG > UAG) or Q70X (UAG > UAG) mutations were challenged with gentamicin in cell-free systems [138]. Higher effects were observed on production of full-length proteins in-vitro for the Q70X variant (~10% of the normal protein) due to the predicted insertion of the wild-type residue, while for the W402X variant (~3%) the original residue (tryptophan) is not predicted in the subset of amino acids introduced by readthrough over UAG PTCs. A functional rescue of around 3% of normal, with reduction of accumulated GAG was measured in cultured fibroblasts compound heterozygous for the two mutations. However, the sequence context of the W402X PTC (UAGG), which has also been challenged with different aminoglycosides, was identified to be low-score in in-vitro assays with a reporter system [73]. On the other hand, the higher susceptibility of the Q70X (UAGC sequence context) to readthrough was also in accordance with theoretical scores [139]. Other MPS I-H nonsense mutations (W180X, Y343X, Q400X and R628X), harbored in the compound heterozygous state, were also challenged, resulting in relevant rescue upon treatment with the exception of the Y343X variant. The highest degree of functional rescue was observed for the R628X/W402X heterozygote, suggesting additive effects of the R628X mutation, whose sequence context (UGAC) is predicted to be highly susceptible to suppression over the unresponsive W402X [57]. Although the re-introduction of arginine on UGA PTCs is predicted to be a low-frequency event, the potential missense change(s) arising from readthrough appeared to be partly tolerated and compatible with residual function. These first evidences prompted other attempts in a knock-in mouse model of MPS I-H harboring the W392X mutation [69,140], corresponding to the frequent W402X variant found in MPS I-H patients, as well as in the presence of NMD attenuators to increase the mRNA levels potentially available as readthrough substrates [141].

In LSDs as models, prediction of inserted amino acids has been tempted as a tool to explain the unsuccessful output of readthrough. Patient fibroblasts harboring the MPS I-H W402X nonsense mutation treated with gentamicin provided further evidence for a partial rescue in a work by Matalonga et al. [67]. As for MPS I-H, the other LSD-causing nonsense mutations challenged for readthrough induction showed similar outputs. The key aspect related to the resulting protein output driven by insertion of a subset of amino acids led authors to provide insights into this issue through in silico predictions. This analysis indicated that missense changes arising from readthrough were potentially tolerated or damaging depending on the type of substitution and localization of the substituted residue, which in turn is driven by the type of PTC. Another attempt to interpret the protein output, albeit performed in a metabolic disorder that does not belong to LSDs, was made for readthrough over 12 nonsense mutations affecting the propionyl-CoA carboxylase enzyme [142]. The investigated nonsense mutations were challenged with aminoglycosides (G418 and gentamicin) or PTC124 (Ataluren) in cellular models with recombinant constructs or in patients’ fibroblasts. The observed protein output in terms of full-length protein production (from 10% to 25% of normal protein) or enzyme activity (highest levels upon G418 treatment) were interpreted in light of the predicted effects through in silico analysis. However, some results were not in accordance with prediction and, at the same time, conflicting predictions were also reported for proteins showing residual levels after readthrough induction.

Additional complexity may emerge when considering oligomeric proteins. Here, the amino acid changes introduced by readthrough may exert unpredicted effects on the quaternary structure of the protein and thus on its biosynthesis/function.

#### 4.1.2. Fabry Disease as a Model for Oligomeric Proteins

Fabry disease (FD, OMIM #301500) is a rare X-linked LSD caused by deficiency of the homodimeric α-galactosidase A (α-gal A) enzyme [143,144,145] and failure in the metabolism of glycosphingolipids. Depending on residual enzymatic activity, FD patients may display either a severe/classic (<1% activity or complete loss-of-function) or a late-onset (residual activity) phenotype [146]. Among the FD-causing mutations in the *GLA* gene, the relatively frequent nonsense mutations show the highest association with the classic FD form.

A first attempt to correct FD nonsense mutations was provided in patients’ fibroblasts bearing the p.R227X (UGA, c.679C > T) variant [67], which resulted in low responsiveness and rescue. These negative results can be explained by the protein context. Indeed, R227 is involved in the formation of the α-gal A active site, in which amino acid substitutions introduced by readthrough are unlikely to be compatible with enzyme function.

A second attempt was made for a wide panel of *GLA* nonsense variants by using G418 as the readthrough-inducing agent [147]. Interestingly, the most responsive variant (p.W209X) in terms of amount of full-length protein revealed a low functional response. In contrast, predicted readthrough-mediated missense variants showed well-detectable levels, normal activity, and correct lysosomal localization. The observed discrepancy in protein/functional levels was not compatible with amino acid insertions as the sole determinant and led to the hypothesis that the interaction between missense monomeric variants and the wild-type monomer might result in potential dominant-negative effects. Noticeably, the wild-type α-gal A protein showed a decreased specific activity, referred as the ratio between functional and protein levels, when co-expressed with readthrough-deriving missense variants at position 209. These findings suggested that, due to the symmetric nature of the α-Gal A homodimer, part of the amino acid changes introduced by readthrough would lead, once full-length proteins are transported into lysosomes, to the assembly of a fraction of functional homodimers as well as heterodimers with lower or no function, resulting from the interaction between wild-type and missense monomeric variants.

### 4.2. Readthrough in Coagulation Factor Disorders

Coagulation factors are involved in a finely tuned series of events (the coagulation cascade) that ultimately leads to the formation of a clot [148]. A simplified view of this complex network of reactions provides that each factor is substrate for the upstream activated enzyme and, at the same time, activates the downstream zymogen within the cascade. In this scenario, a defect in one of these components may lead to a coagulation disorder with different phenotype severity as a function of the residual levels of the affected protein [149].

In the mutational pattern of coagulation factor disorders nonsense mutations are relatively frequent, particularly in the X-linked hemophilia A (HA, OMIM #306700) and B (HB, OMIM #306900), with 10–13% of nucleotide changes resulting in a PTC (http://www.factorviii-db.org; http://www.factorix.org) [150,151,152,153]. Severe disease forms in almost all other bleeding disorders are caused by nonsense mutations associated with alterations of serine proteases, cofactors and inhibitors as well as of proteins involved in primary hemostasis and clot formation/stabilization (http://www.hgmd.cf.ac.uk/ac/index.php).

Nonsense mutations are generally associated with severe forms of bleeding disorders. However, notable exceptions have been reported and characterized for factor (F)VII and FX, in particular for alterations affecting their carboxyl-terminal region, in which missense/nonsense mutations are predicted to have a detrimental impact on secretion of homologous coagulation factors [154,155,156,157,158]. Nonsense variants affecting the carboxyl-terminal region of FX have been shown to have a slight impact on protein secretion/function [159], thus predicting associated asymptomatic phenotypes. On the other hand, the *F7* homozygous p.R462X nonsense mutation, predicting a potentially null genetic condition incompatible with life [160], is associated with an asymptomatic phenotype due to the production of very low amounts of a gain-of-function FVII variant lacking the last five carboxyl-terminal residues [161].

Notably, the low therapeutic threshold sufficient to ameliorate the clinical phenotype in coagulation factor disorders (in the range of 2–5% of normal protein) [149] is compatible with the predicted extent of rescue expected from correction approaches such as readthrough, as well as others targeting mutations at the DNA or mRNA level [162,163,164,165]. This notion drove the first attempts for readthrough as therapy in patients with nonsense mutations in *F8* (HA) and *F9* (HB) [166] as well as *F7* (FVII deficiency, OMIM #227500) [167] genes or in HB mouse models [168]. These studies indicated that the overall response to readthrough was compatible with sub-therapeutic levels or moderate phenotype amelioration for a few nonsense variants. Minimal effects of the drug used (gentamicin), a low suppression efficiency (nucleotide context), and/or amino acid substitutions incompatible with a significant degree of functional rescue (protein context) may help with interpreting these results.

#### 4.2.1. Hemophilia

The interplay between sequence and protein contexts, and thus the impact of inserted amino acids upon readthrough, as well as the influence of the protein context in terms of PTC localization in the coding sequence, has been extensively investigated in in-vitro HB models.

In a first study, a wide panel of recurrent *F9* nonsense mutations was challenged with the readthrough-inducing drug G418. Notably, only two variants (p.W240X and p.R384X) were responsive to G418-promoted suppression, with important indications on the interplay between the key components of nucleotide and protein contexts [169]. Indeed, the relevant functional rescue observed was related to (i) favorable sequence contexts (UGAC and UGAU stop codons for W240X and R384X, respectively), (ii) the re-insertion of the original residue (W204X), and (iii) the gain-of-function features of missense variants predicted to arise from readthrough (R384X). the re-introduction of the original residue, as the driving force for functional rescue of the W240X variant, was confirmed by expression studies with the W240Y variant (devoid of function), resulting from suppression of an UAG codon at the same position, or by the low activity levels (<5% of normal) of naturally-occurring missense changes overlapping (p.W240C [170], p.W240R [171]) or not (p.W240L [172]) with those predicted from readthrough. Overall, these observations indicated that amino acid changes are not tolerated at this FIX position. On the other hand, expression of the most probable missense variant at position 384 (R384W) revealed an overlapping hyperfunctional output, in accordance with the features of the so-called FIX “Padua” position, whose Arg-to-Leu substitution (p.R384L) was found to originate a FIX variant with highly increased specific activity [173].

In a second study, the investigation of three paradigmatic nonsense variants affecting the signal and pro-peptide of FIX (p.G21X, UGA; p.C28X, UGA; p.K45X, UAG) provided experimental evidence for a position-specific effect of PTCs to shape the observed protein output [174]. These two regions are crucial for directing precursor FIX to the endoplasmic reticulum as well as for the key post-translational modification γ-carboxylation [175], and are cleaved intracellularly during FIX maturation. In particular, the relevant rescue of C28X and K45X variants was prevented by specific amino acid sequence constraints, namely the involvement of cysteine at position 28 (between pre- and pro-peptide) and lysine at position 45 (between pro-peptide and mature FIX) in essential cleavage sites. Severe/moderate HB phenotypes associated with different missense mutations at these positions [176,177,178] further supported this interpretation. On the other hand, a relevant protein and functional rescue was observed for the G21X variant, with the noticeable evidence of a specific activity compatible with normal function, suggesting the synthesis of a full-length protein with wild-type features. Strikingly, the corresponding missense variants (bearing tryptophan, cysteine, or arginine) showed a preserved specific activity overlapping that of wild-type FIX. This confirmed that readthrough over the G21X PTC resulted in the synthesis of FIX proteins bearing a subset of amino acids at position 21, but displaying wild-type features after removal of the signal peptide in which missense changes, tolerated in terms of impact on FIX intracellular trafficking/processing and/or structure [179,180], were located. This work provided the first evidence that the favorable localization of PTCs in regions that are removed during protein processing would drive the production of full-length proteins with an intact amino acid sequence, and thus wild-type features, upon readthrough.

The interplay between PTCs responsiveness to readthrough and the investigation of the resulting protein output has been also evaluated for *F8* nonsense mutations causing HA. In this study, Liu and co-workers identified a few high responders to G418-promoted readthrough. This finding was integrated through expression of the predicted missense variants, which revealed that amino acid substitutions at the identified positions were tolerated in terms of secreted protein levels and cofactor activity, thus adding further experimental evidence on the interplay between nucleotide and, in particular, protein contexts [181].

#### 4.2.2. Rare Coagulation Factor Disorders

The combination of nucleotide and protein contexts as determinants of readthrough susceptibility has been also investigated for two homozygous nonsense mutations (p.S112X [182], UGAA; p.C132X [183], UGAC) (http://www.factorvii.org; [184]) in the *F7* gene [185] as well as for one nonsense mutation (p.W461X, UAGA) in the *F10* gene [186]. Importantly, the possibility to rescue protein synthesis and function of proteins such as FVII or FX is of great clinical significance, taking into account that the complete absence of these serine proteases is virtually incompatible with life [160,187] due to their central role within the coagulation cascade. In particular, the functional rescue of these coagulation factors would improve key reaction steps such as the triggering of the whole coagulation process (FVII) or the conversion of prothrombin to thrombin (FX) [188].

The p.S112X and p.C132X nonsense mutations, predicted to produce severely truncated FVII protein devoid of the catalytic site, were identified in two patients affected by severe but not lethal (p.S112X) [182] or unexpectedly moderate (p.C132X) [183] FVII deficiency. Expression studies in the presence or absence of G418 showed a readthrough-mediated increase in secreted protein levels for both FVII nonsense variants, with a parallel increase in the ability of these proteins to restore coagulation. The observed protein output was interpreted through the expression of missense variants, which showed a moderate decrease in secreted and functional FVII levels for missense changes at position 112, whereas secretion and function were completely abolished for changes at position 132. These findings depicted two different scenarios as a function of the nucleotide/protein context considered. Indeed, the different degree of responsiveness to readthrough was in accordance with the susceptibility of PTC contexts (UGAC > UGAA) [57]. On the other hand, amino acid substitutions at position 112, which is surface-exposed, are tolerated and compatible with secretion/function, while at position 132, which is involved in a disulfide bridge essential for FVII structure [189], the only productive event is the insertion of the original cysteine residue [185].

An example of unsuccessful readthrough induction due to protein constraints has been provided for the very rare, and virtually unique, *F10* p.W461X nonsense mutation, found in a compound heterozygote patient with very severe FX deficiency (OMIM #227600). Expression studies on nonsense and missense variants at position 461 showed negligible effects of readthrough induction (G418) as well as a very low functional profile of predicted missense variants, thus pointing toward null features of the p.W461X mutation. This protein output can be interpreted on the basis of the unfavorable UAG sequence context, not predicting re-insertion of the original tryptophan, in combination with protein constraints related to FX function [186]. Indeed, a functional rescue was hampered due to involvement of the amino acid stretch containing the 461 residue in protein-protein interactions with prothrombin [190], impaired by substitutions of the original tryptophan, and thus producing FX variants unable to convert prothrombin to thrombin.

## 5. Conclusions

The results from the above mentioned studies indicate that the insertion of amino acids at a PTC is not a random event, but the suppression of a nonsense mutation is related to the intervention of near-cognate aa-tRNAs able to base pair with the PTC, driving elongation of the growing polypeptide by addition of one among a restricted subset of amino acids. This aspect is of primary importance to predict readthrough-favorable features and the efficacy of this approach to achieve relevant protein/functional rescue, and thus the potential translatability of readthrough as a therapeutic strategy.

The experimental findings on drug-induced readthrough provide a scenario (depicted in Figure 5) in which relevant levels of functional suppression, in terms of correction of protein synthesis and activity, is related to a series of events that may exert complementary or counteracting effects on the resulting protein output, in particular:

(i) the degree of susceptibility of PTC sequence contexts, namely the so-called “leakiness” of PTCs, to be suppressed by the readthrough process itself and by induction of compounds;
(ii) the degree of re-insertion of the original residue within the resulting full-length protein allowing the production of the wild-type polypeptide, which represents the most favorable event;
(iii) tolerated amino acid insertions in terms of protein synthesis, trafficking/secretion and function, or the occurrence of rare improved readthrough-mediated protein outputs driven by gain-of-function features;
(iv) the favorable localization of suppressed nonsense mutations in protein regions, such as signal peptides, that are removed during protein processing and thus outside of the resulting mature protein, an event that is predicted to counteract the potentially negative impact of amino acid substitutions;
(v) potential (dominant-)negative effects of readthrough-derived amino acid insertions on higher-level organization, such as oligomerization, of the target protein.

This complex network of molecular events, resulting in a different degree of functional or productive readthrough, indicates that the nucleotide and protein contexts contribute to restrict the number of responsive PTCs, particularly for proteins with enzymatic activity. On the other hand, this complex and hardly predictable interplay between different molecular determinants might have led to an underestimation of the potency of the available or under-development readthrough-inducing drugs. Nevertheless, the availability of compounds able to efficaciously induce readthrough with relatively low side-effects is a primary goal to achieve for therapeutic purposes.
